# A Double-Teacher Model Capable of Exploiting Isomorphic and Heterogeneous Discrepancy Information for Medical Image Segmentation

**DOI:** 10.3390/diagnostics13111971

**Published:** 2023-06-05

**Authors:** Junguo Zou, Zhaohe Wang, Xiuquan Du

**Affiliations:** 1 School of Information Engineering, Chuzhou Polytechnic, Chuzhou 239000, China; 2 School of Computer Science and Technology, Anhui University, Hefei 230601, China

**Keywords:** semi-supervised learning, left atrium segmentation, isomorphic discrepancy information, heterogeneous discrepancy information

## Abstract

Deep learning, with continuous development, has achieved relatively good results in the field of left atrial segmentation, and numerous semi-supervised methods in this field have been implemented based on consistency regularization to obtain high-performance 3D models by training. However, most semi-supervised methods focus on inter-model consistency and ignore inter-model discrepancy. Therefore, we designed an improved double-teacher framework with discrepancy information. Herein, one teacher learns 2D information, another learns both 2D and 3D information, and the two models jointly guide the student model for learning. Simultaneously, we extract the isomorphic/heterogeneous discrepancy information between the predictions of the student and teacher model to optimize the whole framework. Unlike other semi-supervised methods based on 3D models, ours only uses 3D information to assist 2D models, and does not have a fully 3D model, thus addressing the large memory consumption and limited training data of 3D models to some extent. Our approach shows excellent performance on the left atrium (LA) dataset, similar to that of the best performing 3D semi-supervised methods available, compared to existing techniques.

## 1. Introduction

The ability to accurately segment the left atrial region from heart images plays a key role in the field of medical images. Deep learning has performed well at many tasks to find organs or lesions in medical images because of its efficient use of labeled data. However, the high performance of deep learning relies on a large amount of labeled data, which are difficult to obtain for medical images because accurate annotation can only be done by medical experts with specialized knowledge, which is costly and burdensome. Semi-supervised learning opens up a new way of thinking through its ability to exploit unlabeled data.

Many semi-supervised methods based on consistency regularization have started to appear, with relatively good results. However, the lack of labeled data during training limits their ability to identify key features and hence their accuracy, which leads to bias in the prediction of unlabeled images. The models tend to have large discrepancies in the prediction of features that are difficult to identify. A model will improve the performance if these regions are focused. Therefore, to find these challenging regions and derive some useful features from them is the key to improving model performance. Discrepancy information has an important role in finding challenging regions. However, many semi-supervised methods based on consistency regularization exploit unlabeled data by reducing the discrepancy of prediction to make their predictions consistent, which causes them to exploit inter-model consistency, but not discrepancy. In addition, these methods do not focus on challenging areas. Our method exploits inter-model discrepancy to find these challenging regions and obtain some features from them that are harder to identify but improve model performance. However, finding these regions is not easy because they are usually irregularly shaped, scattered, and highly similar to surrounding regions. For this problem, some methods find these regions by uncertainty maps, and later discard them to improve performance. For example, Monte Carlo dropout [1] was used to compute regions of high uncertainty in teacher model predictions, and regions of high uncertainty were removed so that the student model would learn more accurate data [2]. However, to blindly discard these regions can cause the model to fail to obtain some of their useful features for optimization; hence, these regions must be considered. Our approach looks for and focuses on regions of high uncertainty through the information on discrepancy between models. Other methods have addressed inter-model discrepancy. A recurrent pseudo-labeling scheme was used to convert the prediction differences between the two decoders into unsupervised losses, thus allowing the whole framework to capture some features from challenging regions of unlabeled images, reducing prediction differences between them [3]. Although this approach considers the discrepancy between the models, it does not extract them for further exploitation. This does not guarantee that the model will focus on high-uncertainty regions in the unlabeled images. Additionally, the method only focuses on the discrepancy between isomorphic models, and not between heterogeneous models, which may lead the model to obtain incomplete discrepancy information, and to miss some regions of high uncertainty. Most semi-supervised frameworks use a 3D base model in the left atrial image segmentation task, which, compared to 2D models, usually has more parameters, and requires more memory and longer training times, with less training data [4]. We design a mean teacher model based on a 2D model, with an additional teacher model that can use both 2D and 3D information.

In summary, we design a double-teacher model to accomplish the task of left atrial image segmentation. Our mean-teacher framework encourages both student and teacher models to maintain consistent predictions under the same input with different perturbations, thus enabling them to learn from and optimize each other to obtain more information from unlabeled data. Accordingly, our framework can extract the isomorphic/heterogeneous discrepancy information between the predictions of the student and teacher models, and their combination can represent the uncertainty information between them to some extent. For more accurate extracted discrepancy information, our framework uses labeled images and their corresponding labels to optimize the final predictions of the 2D teacher model. We first supervise the extracted discrepancy information, using a loss function to ensure consistent predictions between the student and teacher models. We further use the extracted discrepancy information to improve the loss of consistency between the two teacher models, so that the model can focus on regions with high uncertainty. Our framework (ISO-MT) can then obtain some features from challenging regions in the images to improve the model prediction results. Our semi-supervised method obtained good results in experiments on the MICCAI 2018 Atrial Segmentation Challenge dataset, with similar performance to the latest 3D model-based semi-supervised framework.

The main contributions of this paper are as follows. First, we adequately extract isomorphic discrepancy information between isomorphic models and heterogeneous discrepancy information between heterogeneous models, and optimize the final prediction of the 2D teacher model using the labeled images and their corresponding labels, thus indirectly optimizing the extracted two types of discrepancy information. Second, we use the extracted discrepancy information to indicate challenging regions, and focus on these by optimizing the inter-model consistency loss to obtain some hard-to-identify features. Third, the proposed methodological framework performs similarly to the state-of-the-art 3D model-based methodological framework in the semi-supervised left atrial segmentation task for the LA dataset.

## 2. Related Work

We describe work related to semi-supervised learning in the left atrial segmentation task and uncertainty.

### 2.1. Semi-Supervised Learning

Semi-supervised learning can be categorized into traditional and deep learning methods. Traditional methods perform the segmentation task by manually designing features, mainly including clustering-based models. Portela et al. [5] proposed a clustering-based semi-supervised classifier to improve the quality of MR brain tissue segmentation. You et al. [6] proposed a priori-based model for retinal vessel extraction based on radial projection and semi-supervised methods, solving the problem of traditional segmentation methods in distinguishing between fine and wide vessels. These traditional methods have some limitations because they are based on handcrafted features. Deep learning methods can be divided into two main categories. Some methods assume that the clusters of each class should be compact and of low entropy. Kalluri et al. [7] proposed a framework to solve the new problem of generic semi-supervised semantic segmentation that accomplishes low-entropy prediction and can reduce deployment costs. Zhang et al. [8] proposed a deep adversarial network (DAN) for the semantic segmentation of biomedical images, consisting of a segmentation network and an evaluation network that distinguishes between the segmentation results of unlabeled and labeled images. A more mainstream approach is to transform the probability map of model predictions into pseudo-labels by using sharpening functions or fixed thresholds, and then make the model learn to generate low-entropy predictions from unlabeled images under the supervision of the pseudo-labels. Bai et al. [9] developed an iterative framework in which the network predicts unlabeled data in each iteration, generates pseudo-labels, refines them by conditional random fields (CRF) [10], and uses them to update the network. Rizve et al. [11] proposed an uncertainty-aware pseudo-label selection (UPS) framework that reduces the amount of noise in the training process to improve the quality of pseudo-labels, and thus the performance of the framework. Zhou et al. [12] proposed deep multiplanar cooperative training (DMPCT) to accomplish abdominal multi-organ segmentation in 3D CT images, which can mine consensus information from multiple planes and realize high performance by generating more reliable pseudo-labels to help in multiplanar fusion training. The second type of method follows the smoothing assumption based on consistency regularization, which means that certain perturbations of the input should produce similar results and not deviate greatly. Wang et al. [13] proposed a semantic data augmentation algorithm to extend the data using semantic rich directions, and applied this to semi-supervised learning to improve the performance of the model, as well as the consistency constraint. Sajjadi, JavanMarti, and Tasdizen [14] proposed a semi-supervised method with stochastic transformation and perturbation regularization to improve performance by minimizing the variance of multi-pass prediction samples. A transform-consistent self-sensing model introduced rotations and flips to encourage consistent prediction of the same input under different regularization conditions for a network under training [15]. Good performance was achieved in the task of skin lesion segmentation. Xie et al. [16] proposed a semi-supervised model based on pairwise relationships that exploits the semantic consistency between each pair of images in the feature space to segment glands in histological images. Luo et al. [17] designed an uncertainty-corrected pyramidal consistency (URPC) regularization framework to learn from meaningful consensus regions at different scales to accomplish total tumor volume (GTV) segmentation to assist in radiotherapy for nasopharyngeal carcinoma (NPC), and realized excellent segmentation performance. Among such methods, self-aware approaches represented by the mean-teacher model [18] have attracted much attention. The model is optimized by making the predictions of the student and teacher models consistent under different disturbances, for the purpose of student learning from the teacher. Yu et al. [2] introduced uncertainty maps calculated by Monte Carlo [1] methods to improve the accuracy of teacher model predictions based on the mean-teacher model. Li et al. [19] introduced a transformation consistency strategy in the mean-teacher model, which was used to enhance the regularization of pixel-level predictions and improve the generalization ability of the model. Luo et al. [20] introduced a dual task in the mean teacher model, predicting the pixel-level segmentation map and the geometric perceptual level set of the target, to achieve consistency in the dual task and substantially improve model performance. Low-entropy models generated based on the clustering assumption and models based on consistency regularization have improved the performance of semi-supervised models, while our approach is designed based on consistency regularization.

### 2.2. Semi-Supervised Image Segmentation of Left Atrium

Semi-supervised methods have been applied successfully to left atrial image segmentation. Many of the more successful such methods are designed based on consistency regularization. Li et al. [21] developed a multitasking deep network capable of jointly predicting surface semantic segmentation of objects and symbolic distance maps (SDMs) for effective left atrial segmentation. The introduction of adversarial losses between the predicted SDMs of unlabeled and labeled data enabled the network to more effectively capture shape perceptual features. Luo et al. [20] did not simply construct a network or data-level perturbations, but achieved task-level regularization by jointly predicting pixel-level segmentation maps and geometric-aware level-set representations of the target, and introduced dual-task consistent regularization between the level-set generated segmentation maps and directly predicted segmentation maps to better segment the left atrium. Yu et al. [2] introduced the mean teacher model [18] to left atrium segmentation, where students were motivated to learn from the teacher by enhancing the consistency of predictions between the student and teacher models in the training model. Constraints on the output of the teacher model ensured the correctness of the learned information, thus improving segmentation performance. Wang et al. [22] proposed a double uncertainty weight based on the mean teacher model semi-supervised segmentation method and applied it to left atrial segmentation. The method uses Bayesian deep learning to train the teacher model to obtain segmentation and feature uncertainty, which can produce more accurate teacher predictions, and thus improve performance. Shumeng Liet et al. [23] designed a hierarchical consistency regularized mean teacher framework that can increase the consistency loss between feature maps, enabling the network to learn diverse information and achieve superior performance in left atrial segmentation. Wu et al. [3] proposed a mutual consistency network (MC-Net) consisting of an encoder and two decoders, where the prediction differences between the decoders are converted to unsupervised loss through circular labeling, which enables the model to capture generalized features from challenging regions in unlabeled images, resulting in consistent and accurate predictions for both decoders. Although the above methods achieve good results at semi-supervised left atrial image segmentation, enough attention is not given to the challenging regions in unlabeled images.

### 2.3. Uncertainty

With the development of deep learning, uncertainty has gained wide attention. In addition to the prediction, reliability is needed. The uncertainty can detect the wrong prediction of the model and calculate the confidence of prediction results. Lakshminarayanan et al. [24] trained multiple predictors, considering that the estimation of different points in the model should differ, and determined the output between multiple predictors by calculating the statistical differences. Gal and Ghahramani [25] developed a theoretical framework that uses dropout training in deep neural networks (NNs) as approximate Bayesian inference in a deep Gaussian process, and proposed to use MC dropout to compute model uncertainty, significantly reducing computational cost. The uncertainty of the model is computed by the prediction differences between submodels. Multiple submodels usually must be trained, which is computationally expensive, while the dropout operation can extract multiple submodels without training them individually, which greatly reduces the computational cost. Yu et al. [2] used the mean teacher model to segment the left atrium, calculating the uncertainty of the teacher model using a Monte Carlo method, to remove the high uncertainty in the teacher model prediction regions, thus providing more accurate predictions for the student learning model. Sedai et al. [26] applied the student teacher model to segment retinal layers. The teacher model was trained by Bayesian deep learning, and could provide soft segmentation labels and uncertainty maps, where the latter can estimate the pixel-level confidence of the segmentation quality of the teacher model. The uncertainty map and soft segmentation labels work together to optimize the student model, improving the performance of the overall model. Wang et al. [22] used Bayesian deep learning to train the teacher model to obtain segmentation and feature uncertainty, with which the teacher model could produce more accurate predictions, thus improving the performance of the overall model. Inspired by the above work, we calculate the inter-model uncertainty by computing the inter-model variance.

## 3. Methods

**Figure 1:**
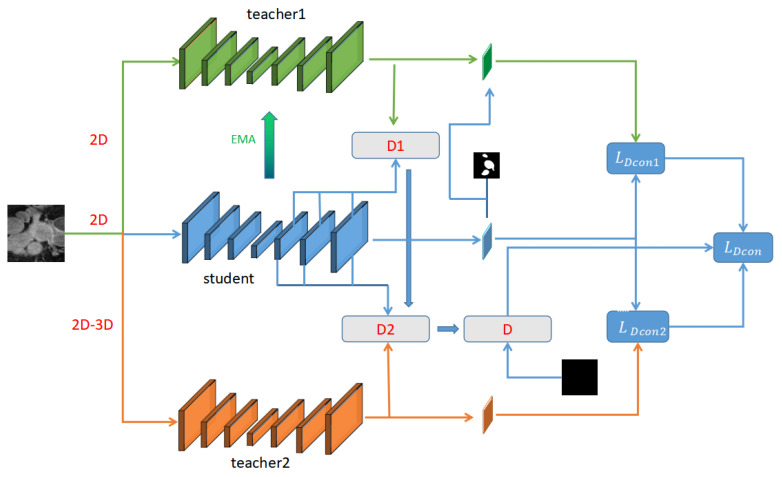
Framework of our approach.

Figure 1 illustrates our proposed mean teacher framework, whose approach follows the basic principles of the mean-teacher framework by taking 2D medical images with different interferences as input, and encouraging consistency in the predictions of student and teacher model segmentation. The 2D student and teacher models have the same network structure. To leverage the unlabeled data for consistency between the student and teacher models and learn the features of challenging regions in images, we extract the isomorphic discrepancy information between the final output of the 2D teacher model and student model, extract the heterogeneous discrepancy information between the final outputs of the teacher that can process both 2D and 3D information and student models, and then the discrepancy information is combined and supervised simultaneously. The isomorphic and heterogeneous discrepancy information extracted from the framework can also optimize the consistency loss between the student and teacher models, allowing the framework to focus more on challenging regions, thus improving performance. We also optimize the final predictions of the 2D teacher models to some extent by having labeled images and their corresponding labels, thus indirectly optimizing the extracted isomorphic and heterogeneous discrepancy information, which is equivalent to indirectly optimizing the whole framework.

### 3.1. Supervised Segmentation

In our framework, the networks for the student model and 2D teacher model are constructed based on 2D Unet [27]. To fully extract the difference information between the student and teacher models, we add a 1 × 1 convolutional layer and sigmoid layer to the last upsampling layer of the 2D Unet network decoder, and an upsampling layer, 1 × 1 convolutional layer, and sigmoid layer to the penultimate upsampling layer, to obtain an intermediate prediction map of the same size as the final prediction map. The teacher model fuses 2D and 3D convolution [28]. We feed the labeled dataset $D_{l}={\left\{\left(x_{i},y_{i}\right)\right\}}_{i=1}^{L}$, where $x_{i}\in R^{H\times W}$ is the input image and $y_{i}\in {\left\{0,1\right\}}^{H\times W}$ is the 2D ground truth, to the student model for training, aiming to minimize the supervised loss function,

(1) $$l_{\sup\;}=\sum_{i=1}^{L}l_{\mathrm{dice}\;}\left(f_{s}\left(x_{i};\theta \right),y_{i}\right),$$

where $l_{\sup\;}$ is the Dice loss function to evaluate the network prediction quality on the labeled data input, $f_{s}\left(\cdot \right)$ is the network weight of the student model, and L is the number of labeled images in the input.

### 3.2. Mean Teacher Framework with Two Teachers

For a semi-supervised segmentation task, the data used for training usually have two components, labeled data $D_{l}={\left\{\left(x_{i},y_{i}\right)\right\}}_{i=1}^{L}$, where $x_{i}\in R^{H\times W}$ is the input image and $y_{i}\in {\left\{0,1\right\}}^{H\times W}$; and unlabeled data $D_{u}={\left\{\left(x_{i},y_{i}\right)\right\}}_{i=L+1}^{L+U}$, where $x_{i}\in R^{H\times W}$ is the input 2D slice and $y_{i}\in {\left\{0,1\right\}}^{H\times W}$ is the 2D ground truth. L represents the number of labeled 2D image slices, and U represents the number of unlabeled 2D image slices. In our mean teacher framework, the input to the 2D teacher is the unlabeled 2D slices, while the input to the teacher model can handle both 2D and 3D information, and the 3D slices are obtained by combining the three 2D slices adjacent to the front and back of the 2D slices.

The prediction results of the teacher model in the mean teacher model need to be of higher quality than the student model to provide better guidance to students. Recent studies [18,29] have shown that if the model merges the network prediction results from different training processes, it can improve the quality of the prediction of the target and therefore be used to improve the performance and prediction results of the teacher model. Accordingly, the student model and the 2D teacher model share the same network architecture, and the weights of the teacher model are updated using the exponential moving average (EMA) of the weights of the student model. The weights of the teacher model at step t are updated as $\theta {}_{t}^{T}=\eta \theta_{t-1}^{T}+\left(1-\eta \right)\theta_{t}^{S}$, where η is used to control the update rate of the EMA, which is the respective ratio of the weights of the student model at step t and the weights of the teacher model at step t − 1 to the weights of the teacher model at step t. The teacher model in our framework can handle both 2D and 3D information to further guide the segmentation of the student model. To ensure that the predictions of the 2D teacher model can give more accurate guidance to the student model, we use the labeled data processed by the student model in the same input to optimize the prediction results of the 2D teacher model [30]. The steps are as follows: 1. We multiply the prediction results of the student model by its corresponding labels to obtain a new batch of images $S_{i\;}$ that contain only the prediction results of the student model for the target region,

(2) $$S_{i}=f_{s}\left(x_{i};\theta \right)\times y_{i};$$

2. The average predicted probability A of the student model for the target region of this batch of labeled images is calculated as

(3) $$A=\frac{\mathrm{Sum}\left(S_{i}\right)}{\mathrm{Sum}\left(y_{i}\right)},$$

where Sum is the sum of the pixel values of the image;

3. A is the average predicted probability of the target region of this batch of labeled images by the student model. Since the data input into the framework come from the same dataset each time, the features of the targets have some similarity. Each time a batch of data is input into the framework, the average predicted probability A is obtained, which indicates the lowest probability of the pixel that is most probably the target predicted by the student model in this batch of input labeled images. This can guide the 2D teacher model to optimize its own prediction. In the prediction results of the 2D teacher model for the same batch of unlabeled data, when the predicted probability is greater than or equal to A, then the pixel is most probably the target, and when the predicted probability is less than A, the pixel is most probably the background, and the prediction results of the 2D teacher model are optimized in this way. For the unlabeled data, we want the segmentation results of the student model and the two teacher models to be consistent and valid. To achieve consistency between the student and the two teacher models, we first extract the discrepancy information (D1, D2) between the last output of the student model, the output of the last upsampling layer, and the output of the penultimate upsampling layer and the last output of the two teacher models, and combine the obtained discrepancy information to obtain discrepancy information D. Then, we use all-zero-valued images and D to compute our discrepancy loss to constrain the consistency between the student and teacher models and to motivate them to learn from each other. Additionally, during the calculation of D1, the predictions of the 2D teacher model are optimized by A, as follows.

For the student and 2D teacher models, the discrepancy information $D_{1h\;}$ between the high-confidence target regions is first calculated on the basis of the average probability value A,

(4) $$f_{t1h}=f_{t1}\left(x_{i};\theta^{\prime },\xi^{\prime }\right)>=A$$

(5) $$\begin{array}{c} D_{1h}=\sum_{i=L+1}^{L+U}a∥f_{s}\left(x_{i};\theta ,\xi \right)-f_{t1}\left(x_{i};\theta^{\prime },\xi^{\prime }\right)\times f_{t1\;h}∥\\ +b∥f_{s1}\left(x_{i};\theta ,\xi \right)-f_{t1}\left(x_{i};\theta^{\prime },\xi^{\prime }\right)\times f_{t1\;h}∥\\ +c∥f_{s2}\left(x_{i};\theta ,\xi \right)-f_{t1}\left(x_{i};\theta^{\prime },\xi^{\prime }\right)\times f_{t1\;h}∥. \end{array}$$

Then, the discrepancy information $D_{1l\;}$ between low-confidence target regions is calculated,

(6) $$f_{t1l}=f_{t1}\left(x_{i};\theta^{\prime },\xi^{\prime }\right)<A$$

(7) $$\begin{array}{c} D_{11}=\sum_{i=L+1}^{L+U}a∥f_{s}\left(x_{i};\theta ,\xi \right)-f_{t1}\left(x_{i};\theta^{\prime },\xi^{\prime }\right)\times f_{t1l}∥\\ +b∥f_{s1}\left(x_{i};\theta ,\xi \right)-f_{t1}\left(x_{i};\theta^{\prime },\xi^{\prime }\right)\times f_{t1l}∥\\ +c∥f_{s2}\left(x_{i};\theta ,\xi \right)-f_{t1}\left(x_{i};\theta^{\prime },\xi^{\prime }\right)\times f_{t1l}∥. \end{array}$$

Combining $D_{1h\;}$ and $D_{1l\;}$ gives the discrepancy information $D_{1}$ between the student and 2D teacher models,

(8) $$D_{1}=HighD_{1h}+LowD_{1l}.$$

It is straightforward to calculate the discrepancy information $D_{2\;}$ between the student model and teacher model that can handle both 2D and 3D information,

(9) $$\begin{array}{c} D_{2}=\sum_{i=L+1}^{L+U}a∥f_{s}\left(x_{i};\theta ,\xi \right)-f_{t2}\left(x_{i};\theta^{\prime },\xi^{\prime }\right)∥\\ +b∥f_{s1}\left(x_{i};\theta ,\xi \right)-f_{t2}\left(x_{i};\theta^{\prime },\xi^{\prime }\right)∥\\ +c∥f_{s2}\left(x_{i};\theta ,\xi \right)-f_{t2}\left(x_{i};\theta^{\prime },\xi^{\prime }\right)∥. \end{array}$$

The final discrepancy information is

(10) $$D=mD_{1}+nD_{2}.$$

Based on the obtained discrepancy information D, the discrepancy loss is calculated as

(11) $$l_{D}=l_{MSE}\left(D,\;\mathrm{Black}\right).$$

The outputs of $f_{s}\left(\cdot \right)$, $f_{s1}\left(\cdot \right)$, and $f_{s2}\left(\cdot \right)$ are, respectively, the outputs of the segmentation network, last upsampling layer, and penultimate upsampling layer of the student model segmentation network. $f_{t1}\left(\cdot \right)$ denotes the segmentation network of the 2D teacher model, and $f_{t2}\left(\cdot \right)$ is the network of the teacher model that can process both 2D and 3D information. The inputs $f_{t1h}\left(\cdot \right)$ and $f_{t1l}\left(\cdot \right)$, respectively, denote the high- and low-confidence target regions. $l_{\mathrm{MSE}}$ is the mean square error loss; θ is the network weight of the student model; $\theta^{\prime }$ is the network weight of the teacher model; ξ is the network perturbation of the student model; $\xi^{\prime }$ is the network perturbation of the teacher model; a, b, c, High, Low, m, and n are constant coefficients; and Black is all images with zero values of the same size as D. The extracted difference information (D) can represent the uncertainty of our overall framework, and we wish to focus on regions with high difference information, i.e., with high uncertainty; hence, we designed a new consistency loss function for the student model and the two teacher models, which can make the overall framework pay more attention to regions with high uncertainty during training; as such, as many useful features as possible can be captured from regions with high uncertainty and thus improve segmentation performance. Our consistency loss is calculated as follows. The loss $l_{\mathrm{Dcon}1}$ of consistency between the student model and the 2D teacher model is calculated according to D as

(12) $$\begin{array}{c} l_{\mathrm{Dcon}1\;}=\sum_{i=L+1}^{L+U}\alpha l_{\mathrm{MSE}}\left(\mathrm{II}\left(D>TH\right)f_{s}\left(x_{i};\theta ,\xi \right),\mathrm{II}\left(D>\mathrm{TH}\right)f_{t1}\left(x_{i};\theta^{\prime },\xi^{\prime }\right)\right)\\ +\beta l_{\mathrm{MSE}}(\mathrm{II}(D<\left.=Th)f_{s}\left(x_{i};\theta ,\xi \right),\;\mathrm{II}\;\left(D<=\mathrm{TH}\right)f_{t1}\left(x_{i};\theta^{\prime },\xi^{\prime }\right)\right). \end{array}$$

The loss of consistency between the student model and the teacher model that can handle both 2D and 3D information is calculated as

(13) $$\begin{array}{c} l_{\mathrm{Dcon}2\;}=\sum_{i=L+1}^{L+U}\alpha l_{\mathrm{MSE}}\left(\mathrm{II}\left(D>TH\right)f_{s}\left(x_{i};\theta ,\xi \right),\mathrm{II}\left(D>\mathrm{TH}\right)f_{t2}\left(x_{i};\theta^{\prime },\xi^{\prime }\right)\right)\\ +\beta l_{\mathrm{MSE}}(\mathrm{II}(D<\left.=Th)f_{s}\left(x_{i};\theta ,\xi \right),\;\mathrm{II}\;\left(D<=\mathrm{TH}\right)f_{t2}\left(x_{i};\theta^{\prime },\xi^{\prime }\right)\right). \end{array}$$

We combine $l_{\mathrm{Dcon}1}$ and $l_{\mathrm{Dcon}2}$ to obtain

(14) $$l_{Dcon}=wl_{Dcon1}+vl_{Dcon2},$$

where II(·) is the indicator function, which plays the role of judgment; TH is the threshold coefficient of the selected region; and α, β, w, and v are constant coefficients. We combine supervised loss $l_{\sup\;}$, variance loss $l_{D\;}$, and improved consistency loss $l_{\mathrm{Dcon}\;}$ to obtain

(15) $$l_{total}=Xl_{sup}+Yl_{D}+Zl_{Dcon},$$

where X, Y, and Z are constant coefficients controlling the ratio between the individual losses.

## 4. Experimental Analysis

We describe the following aspects: Data set and data pre-processing; Experimental details; Evaluation Metrics; Comparison with other methods; Ablation experiments; Relationship between mean discrepancy rate and mean dice; Exploring the importance of uncertainty derived from discrepant information; Exploring the importance of teacher models that can handle both 2D and 3D information; and Clinical applications.

### 4.1. Dataset and Preprocessing

We evaluated our semi-supervised segmentation method on the dataset from the 2018 Left Atrial Segmentation Challenge [31], consisting of 100 3D gadolinium-enhanced magnetic resonance imaging scans (GE MRIs) and LA segmentation masks. These scans have an isotropic resolution of 0.625 × 0.625 × 0.625. Based on previous work on this dataset [2,3,20,21,22,32], we used 80 of these 100 scans for semi-supervised training, and 20 for testing. Because our semi-supervised method uses 2D images, we further sliced the scanned 3D images in the Z-axis; and to further enhance the training effect, we performed a simple brightening operation on the obtained slices, while removing invalid images without target regions. The image used in our semi-supervised framework is composed of slices and their neighbors, and is not a complete 3D image.

### 4.2. Experimental Details

Our framework was implemented in PyTorch using an NVIDIA 3060 GPU. For the optimization update of the network parameters, we used an SGD optimizer (weight decay = 0.0001, momentum = 0.9), and the learning rate of the SGD optimizer was set to 0.02. To obtain the best performance, we optimized the framework for about 5000 iterations. The batch size was 8, and each batch consisted of four labeled 2D image slices and four unlabeled 2D image slices. The processed 3D slices corresponding to the four unlabeled 2D image slices were also fed into the frame, which accepted 2D image slices of size 128 × 128. The decay coefficient η of the exponential moving average (EMA) in our framework was set to 0.99 [2]. All experiments were performed in the same setting with a fixed random seed (software: PyTorch 1.8.0+cu11.1 and Python 3.8).

### 4.3. Evaluation Metrics

We used Dice, Jaccard, mean surface distance (ASD), and 95% Hausdorff distance (95hd) to evaluate performance on this left atrial dataset. These are defined as

$$\mathrm{Dice}\;=\frac{2\times \mathrm{TP}}{\mathrm{FN}+\mathrm{TP}+\mathrm{TP}+\mathrm{Fp}}$$

$$\;\mathrm{Jaccard}\;=\frac{\mathrm{TP}}{\mathrm{FN}+\mathrm{TP}+\mathrm{Fp}}$$

$$ASD=\frac{1}{\left|X|+|Y\right|}\left(\sum_{x\in X}d\left(x,Y\right)+\sum_{y\in Y}d\left(y,X\right)\right)\;d\left(x,Y\right)=\min_{y\in Y}{∥x-y∥}_{2}$$

95HD=max[d(X,Y),d(Y,X)]×95%

where TP, TN, FP, and FN are true positive, true negative, false positive, and false negative, respectively; X and Y are predicted and labeled boundaries, respectively; d(X,Y) is the largest distance from a point on X to the nearest point on Y; x denotes the coordinates of a point on the boundary of the predicted result; and y denotes the coordinates of a point on the boundary of the label.

### 4.4. Comparison with Other Methods

This dataset has two popular semi-supervised experimental setups: 10% labeled data and 90% unlabeled data, and 20% labeled data and 80% unlabeled data. We performed experimental validation under both. Table 1 shows the quantitative results of our method (ISO-MT) and other advanced semi-supervised methods, such as the Uncertainty-Aware Mean Teacher model (UA-MT) [2], Shape-Aware Semi-Supervised method (SASSNet) [21], double uncertainty weighted semi-supervised method (DUWM) [22], local and global-based structure-aware entropy regularized averaging teacher model (LG-ER-MT) [32], semi-supervised segmentation method using dual task consistency (DTC) [20], and semi-supervised segmentation method using mutual consistency (MC-Net) [3]. On this left atrial segmentation dataset, all these recent 3D semi-supervised segmentation methods achieved good performance, with DTC relying on dual-task consistency achieving 86.57% and 89.42% Dice similarity coefficients at 10% and 20% labeling experimental settings, respectively; and DUWM achieving 86.57% and 89.42% in dual uncertainty based on the teacher–student model (segmentation uncertainty, feature uncertainty) weighted semi-supervised segmentation method achieved 85.91% and 89.65% Dice performance. UA-MT, SASSNet, and LG-ER-MT relied on uncertain self-aware, shape-aware, local, and global structure-aware entropy regularization to achieve 84.25% and 88.88%, 87.32% and 89.54%, and 85.54% and 89.62% Dice similarity coefficients in 10% and 20% labeled experimental settings, respectively. Among these 3D semi-supervised methods, MC-Net achieved the best Dice similarity coefficients of 87.71% and 90.34% in the 10% labeled data and 20% labeled data experimental settings, respectively, and several other evaluation metrics by virtue of reducing the discrepancy information between different encoders for generalized feature representation of challenging regions in unlabeled images. In contrast, our two-dimensional semi-supervised method (ISO-MT) achieved the goal of extracting some useful features in challenging regions of unlabeled images by extracting both isomorphic discrepancy information and heterogeneous discrepancy information between student and teacher models, and giving extra attention to regions with large discrepancy information, i.e., with high uncertainty, in unlabeled images. Thus, our framework obtained slightly better Dice similarity coefficients than MC-Net in the experimental setups with 10% and 20% labeled data, 87.93% and 90.85%, respectively. However, since our method (D-MT) is designed based on a 2D network, 3D data play an auxiliary role, and hence it runs faster and consumes less memory than 3D network-based semi-supervised segmentation methods. Furthermore, because our method requires 2D images, more training images can be obtained; notably, one 3D image can yield dozens of 2D image slices. Thus, our method (ISO-MT) still has some advantages.

**Figure 2:**
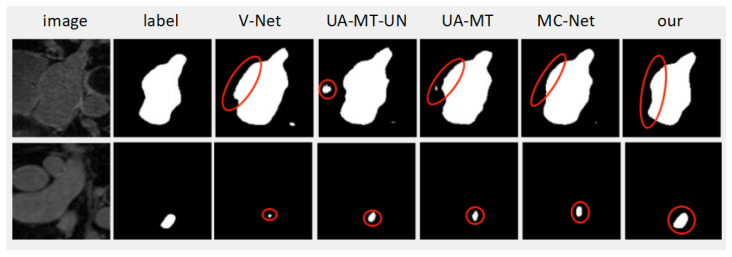
Visualization comparison. Parts circled in red represent prediction results of different methods for challenging regions in image.

Figure 2 shows, from left to right, a visualization of the left ventricular images and their corresponding labels, as well as the prediction results of V-Net, UA-MT-UN, UA-MT, MC-Net, and our method (ISO-MT). All prediction images in Figure 2 are generated by the models of the corresponding methods trained with 20% labeled images. The visualization of the two images is shown in the figure to compare the results. It can be seen that for V-Net, the most basic 3D framework, which is a supervised method relying on a large number of labeled images, the performance of the model is not high when only a small number of labeled images are involved in the training; thus, for the two images in Figure 2, the accuracy of the prediction results obtained by V-Net has a large deviation, especially in the boundary part of the target, and the recognition of small targets is poor. UA-MT-UN and UA-MT are semi-supervised frameworks based on the same mean teacher design, but the teacher model in UA-MT is also exposed to images with labels, just without the use of labels, while the teacher model in UA-MT-UN is only exposed to images without labels. There is a slight difference in the exposed data; the performance of the two images is very close and both discard challenging regions during training, which leads to less favorable prediction results for images with more difficult features (e.g., edges, small targets), as can be seen from the prediction results of the two images in Figure 2. In the first image, UA-MT-UN predicts the target boundary better than UA-MT, but it predicts a larger area in the non-target region. In the second image, the target is smaller, and UA-MT-UN performs better than UA-MT. This method of MC-Net considers these challenging areas; it can be seen from the figure that MC-Net can handle challenging areas, such as target edges and small target areas. Therefore, MC-Net provides better target edges than UA-MT-UN and UA-MT for the predicted image. However, MC-Net is still not good enough. For example, in the prediction results of the first image, the edges are still not accurately predicted, and in the second image, the targets are smaller and the prediction results of MC-Net are not satisfactory enough. Additionally, the prediction results of these two images are not much better than those of UA-MT and UA-MT-UN overall because the incomplete search for challenging regions and the lack of attention to challenging regions lead to less-than-optimal prediction of challenging regions by MC-Net, which in turn leads to less-than-optimal prediction of small target regions in the second image. Our method performs relatively well on these two images because it extracts more complete challenging regions by extracting isomorphic and heterogeneous discrepancy information and pays attention to these regions. In the first image, the edge part of the prediction results of our method framework does not differ much from the labels. In the second image, the prediction of our method for the small target regions is likewise more accurate and more similar to the labels. In conclusion, our method (ISO-MT) can deal with challenging regions in images.

### 4.5. Ablation Experiments

We performed ablation experiments to validate each part of our method, with results as shown in Table 2, from which we see that when only teacher1 works properly in the whole framework, teacher1 is the 2D teacher model. Here, our framework can only extract the isomorphic discrepancy information between the models, and uses this to optimize the consistency loss between the student model and teacher1. Here, since only isomorphic discrepancy information is available, the discrepancy information is not sufficiently complete, causing the framework to focus on challenging regions that are also incomplete and leading to Dice similarity coefficients that are not particularly high in either the 10% or 20% experimental setting, with respective values of 86.81% and 90.19%. Then, our framework can use the labeled data processed by the student model in the same input to optimize the prediction results of teacher1, thus improving the accuracy of extracted isomorphic discrepancy information, which increases the Dice similarity coefficients in the 10% and 20% labeled image experimental settings to 87.75% and 90.65%, respectively. This indicates that the added prediction optimization is useful. If we add teacher2, a teacher model that we designed to handle both 2D and 3D slices, our framework can extract both the isomorphic discrepancy information between the teacher1 and student models and the heterogeneous discrepancy information between the teacher2 and student models. Our method can then combine the isomorphic and heterogeneous discrepancy information to obtain more complete information, and use this to optimize the consistency loss between the student model and both teacher1 and teacher2, which can focus on more challenging regions and improve model performance. The Dice similarity coefficients improve to 87.70% and 90.44% for 10% and 20% labeled images, respectively. Our framework is complete if the prediction optimization is added to the base of teacher1 and teacher2, which can use the labeled data processed by the student model in the same input to optimize the prediction results of teacher1. Here, our framework can extract more complete and accurate discrepancy information, and focus more accurately on those challenging regions, improving model performance. The Dice similarity coefficients improve to 87.93% and 90.85%, respectively. The above analysis shows that both teacher2 and the prediction optimization of our framework are useful, and can play roles in the training and optimization of the entire framework.

**Figure 3:**
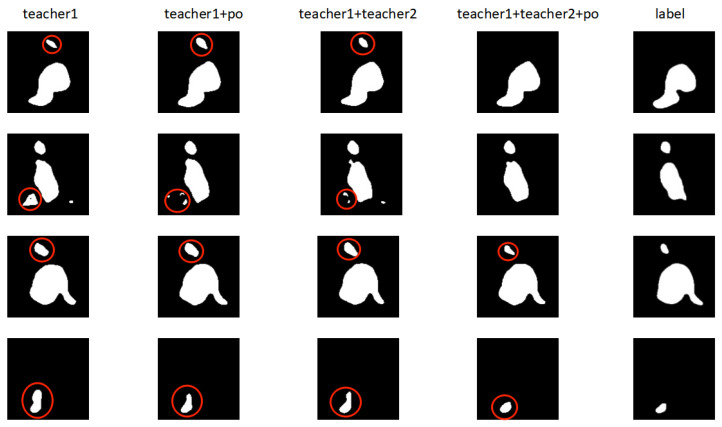
Visualization comparison of ablation experiments. Red circles represent the model’s prediction of challenging regions.

Figure 3 compares some results of the ablation experiment, from left to right: prediction results of the model with only teacher1 in the frame; prediction results of the model with teacher1 and prediction optimization in the frame; prediction results of the model with teacher1 and teacher2 in the frame; prediction results of the model with teacher1, teacher2, and prediction optimization in the frame; and the corresponding labels of the images. From the figure, it can be seen that adding teacher2 or prediction optimization to teacher1 can improve prediction results, reduce the probability that the model identifies non-targets as targets, and improve the accuracy of the prediction results. For the images shown in the figure, the model with only teacher1 in the framework predicted some non-target regions as targets. These should be challenging regions, and the framework with only teacher1 cannot find these completely; hence, the model cannot comprehensively focus on these regions or obtain enough useful information, thus leading to incorrect prediction. It can also be seen that adding either teacher2 or prediction optimization on top of teacher1 can mitigate this situation, but sometimes does not improve the prediction results and reduce the size of non-target regions with prediction errors, in which case adding both teacher2 and prediction optimization may improve the prediction target and reduce the extent of non-target regions with prediction errors. It can also be seen that adding either teacher2 or prediction optimization can optimize the model prediction results, and the simultaneous operation of teacher1, teacher2, and prediction optimization can continue to optimize them. In summary, both teacher2 and prediction optimization are important components in the framework.

### 4.6. Relationship between Mean Discrepancy Rate and Mean Dice

To describe the size of the discrepancy, we use the discrepancy rate Dr, i.e., the ratio of the pixel sum calculated by the student and teacher model, $P_{D\;}$, to that of an image with the same size but with all pixel values of 1, $P_{\mathrm{total}\;}$,

$$\mathrm{Dr}=\frac{p_{D\;}}{P_{\mathrm{total}\;}}.$$

**Figure 4:**
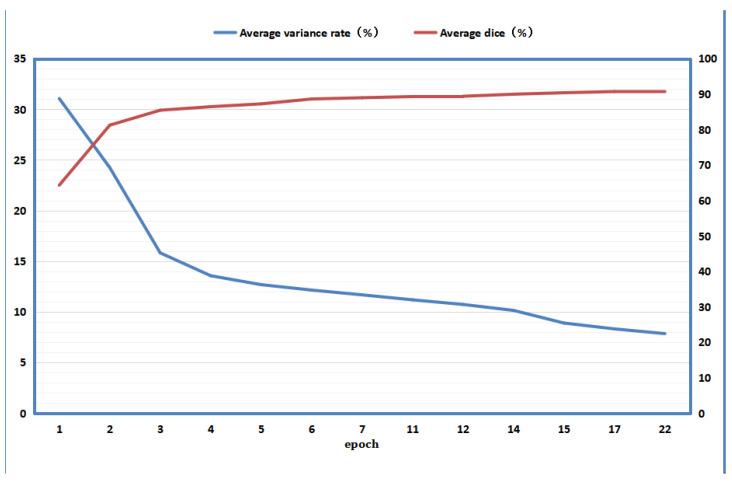
Relationship between mean difference rate and mean Dice.

This can accurately reflect the magnitude of the discrepancy information. During the model training process, discrepancy information can be generated for each iteration, and the discrepancy rate can be calculated. We used Dr to further compute the average discrepancy rate for each iteration of the training process, and calculated the average Dice of the corresponding model on the test set. Figure 4 shows their relationship.

It can be seen from Figure 4 that with increasing training epochs, the discrepancy rate decreases, which indicates less information about the discrepancy and that students and teachers are learning from each other to reduce the discrepancy. Additionally, as the discrepancy rate and information decrease, the average Dice similarity coefficient of the model on the test set increases, which means that the model becomes better. This indicates that the student and teacher models reduce the discrepancy between each other by learning from each other while reducing discrepancy information, and learn useful information and improve their own performance. It can also be seen that the rate of discrepancy reduction decreases with training epochs because, when the discrepancy information is large, it represents performance that is not particularly high when more useful information can be obtained through the discrepancy information to optimize the model, thus making it more accurate and leading to a larger decrease in the discrepancy rate. When the discrepancy rate is relatively small, the performance of the model should be higher, the discrepancy information is less, and the useful information that the model can obtain from it is relatively limited, so the rate of reduction will decrease. It can also be seen that when the training is late, the discrepancy rate decreases very slowly, but the average Dice similarity coefficient still slowly increases, indicating that when the discrepancy information is small, the model in our framework can still obtain some useful optimization information. Figure 4 shows that with increasing epochs, the rate of reduction of the discrepancy rate decreases, and it is already difficult to continue to decrease at the back. Because the magnitudes of the discrepancy loss and discrepancy rate are closely related, the discrepancy loss should not be too large in this case. However, even in this case, the average discrepancy rate of the model continues to rise. This indicates that in the later stage of model training, when the discrepancy information obtained by extracting isomorphic and heterogeneous discrepancy information between models is difficult to reduce, our improved consistency loss due to discrepancy information can improve model performance by focusing on the challenging regions indicated by the discrepancy information. Thus, in Figure 4, when the discrepancy rate is difficult to reduce, the useful information on which the model performance improvement depends is likely to come from the challenging regions indicated by the discrepancy information.

### 4.7. Importance of Uncertainty Derived from Discrepancy Information

**Figure 5:**
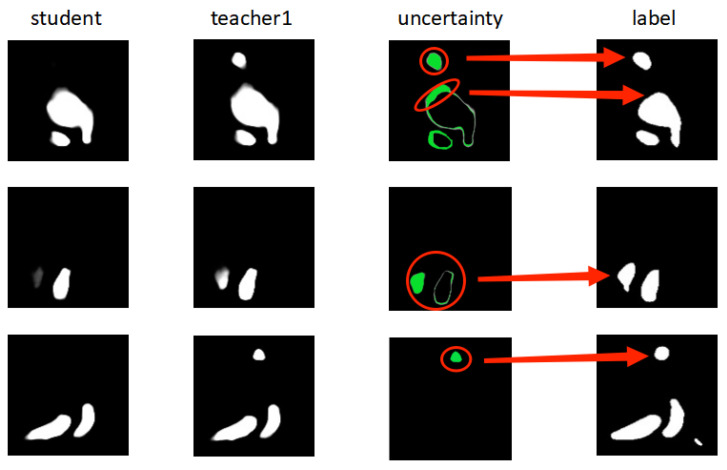
Uncertainty obtained through difference in predictions between student and teacher1. Part of red circle to left of red arrow represents visualization of discrepancy information obtained by student and 2D teacher models; red arrow points to area in label corresponding to discrepancy information.

Figure 5 shows, from left to right, the final prediction results of the student model, final prediction results of the 2D teacher model, approximate uncertainty visualization obtained by calculating the discrepancy between the final prediction results of the student and 2D teacher models during training, and labels of the images. As shown in the figure, for the three images shown, there are some gaps between the prediction results of the student model and the labels, and these are the regions where the prediction performance of the student model is poor, which can also be regarded as the challenging regions, i.e., those with higher uncertainty.

For the first image, the challenging regions are a separate smaller target region at the top and the edge region of the large target, both of which are often difficult areas for image segmentation. This is easy to understand because features near the target boundary and in the smaller target region are more difficult to identify and distinguish. This is also true for the second and third images, where the challenging regions are concentrated at the target boundary and in small target regions. As can be seen from the third column, the uncertainty visualization map obtained by calculating the discrepancy between the prediction results of the student and teacher models can more accurately target the challenging regions, and the high-uncertainty regions correspond to the challenging regions. It can also be seen that for the three images, the prediction results of the teacher model are better than those of the student model, which are closer to the labels. This implies that the teacher model can indeed serve as a guide for the student model in some cases. As can be seen from the above, the uncertainty calculated from the discrepancy between the models can roughly reflect the uncertainty of the images, and is meaningful in roughly locating challenging regions. Since this uncertainty is obtained by calculating the discrepancy between models, it is called discrepancy information; this information helps improve the performance of the overall framework.

### 4.8. Importance of Teacher Models That Can Handle Both 2D and 3D Information

**Figure 6:**
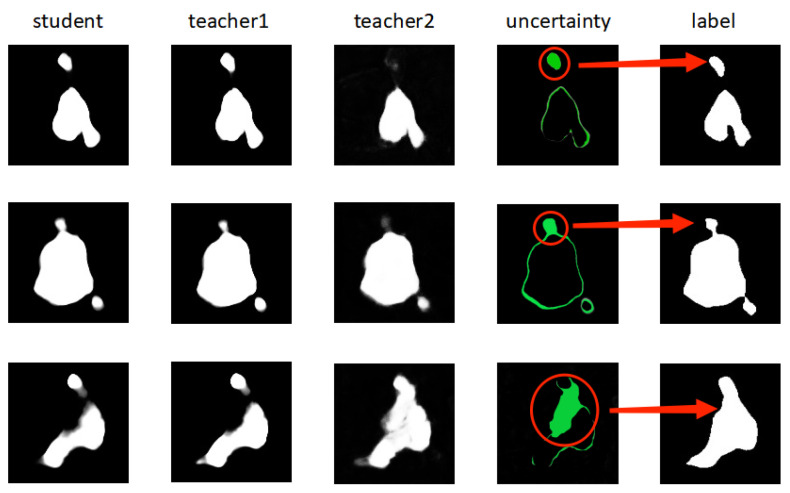
Uncertainty obtained through difference in predictions between students and teacher2. Part of red circle to left of red arrow represents visualization of discrepancy information obtained by student model and teacher model fusing 2D and 3D information; red arrow points to area in label corresponding to discrepancy information.

Figure 6 shows, from left to right, the prediction results of the student and 2D teacher models, the final prediction results of the teacher model that can handle both 2D and 3D information, the approximate uncertainty visualization obtained by calculating the discrepancy between the final prediction results of the student and teacher models that can handle both 2D and 3D information during training, and the labels of images.

As shown in Figure 6, for the three images, the discrepancy between the final prediction results of the student and 2D teacher model is not enormous, which indicates that discrepancy information obtained in such a manner may be insignificant, and the challenging regions that can be targeted are very limited. Here, the final prediction results of the teacher model that can process both 2D and 3D information have a relatively large discrepancy with the prediction results of the 2D teacher model. For the third image, the final prediction results obtained by the teacher model that can process both 2D and 3D information are even closer to the labels of the images, perhaps because the teacher model considers some 3D information, and thus obtains better prediction results than the 2D teacher model. Therefore, for these three images, if the discrepancy between the final predictions of the student and teacher models that can handle both 2D and 3D information is obtained, then this will be richer, and more challenging areas can be targeted, thus improving the performance of the framework. In summary, a teacher model that can handle both 2D and 3D information makes sense, and is useful for improving the performance of the overall framework.

### 4.9. Clinical Applications

Our technique achieves the task of left atrial segmentation and can be used in clinical situations to assist in the treatment of atrial fibrillation, a common cardiac arrhythmia, which refers to the irregular beating of the heart. Surgical treatment of the left atrium can reduce or eliminate AF by preventing the propagation of abnormal electrical signals within the atria. Our technology can be used to segment the left atrium from the patient’s heart image before its surgical treatment, to more precisely determine its location, and thus assist the physician. The steps are as follows: The doctor sends the 3D image of the patient’s heart into our program, whose preprocessing part slices it into some 2D slices that meet its size requirements. The processed 2D slices are fed into our test model to obtain the segmentation results, and the program combines all the slices to form a 3D segmentation result, which is output to the physician for viewing, to assist in locating the left atrium.

## 5. Conclusions

In this paper, we proposed a semi-supervised left atrial segmentation framework (ISO-MT) based on a 2D model. The key idea of our framework was to extract the isomorphic discrepancy information between the prediction results of the student model and 2D teacher model for unlabeled images as well as the heterogeneous discrepancy information between the prediction results of the student model and the teacher model that can process both 2D and 3D information. Isomorphic discrepancy and heterogeneous discrepancy information could be combined as uncertainty information to optimize the consistency between the student model and the two teacher models’ loss, which could better focus the optimized consistency loss on the challenging regions in the unlabeled images; thus, some useful features could be captured from these regions to optimize our method framework. Our (ISO-MT) framework performed similarly to state-of-the-art 3D semi-supervised methods on the left atrial dataset. Our framework was based on a 2D model, so it ran faster, consumed less memory, and obtained relatively more training data for the same number of training images. Our method had some clinical value, was relevant to the diagnosis and treatment of atrial fibrillation, and could assist in the localization of the left atrium, reducing the burden on physicians. Because our method is computerized, there will always be errors. Hence, it cannot achieve 100% accuracy or completely replace the physician in the task of localizing the left atrium.

## Figures and Tables

**Table 1:** Performance comparison of metrics between ours and other methods, where v stands for voxel.

Method	# Scans Used		Metrics			
	Labeled	Unlabeled	Dice	Jaccard	95HD (v)	ASD (v)
V-Net	8 (10%)	72	79.99%	68.12%	21.11	5.48
UA-MT [2]	8 (10%)	72	84.25%	73.48%	13.84	3.36
SASSNet [21]	8 (10%)	72	87.32%	77.72%	9.62	2.55
DUWM [22]	8 (10%)	72	85.91%	75.75%	12.67	3.31
LG-ER-MT [32]	8 (10%)	72	85.54%	75.12%	13.29	3.77
DTC [20]	8 (10%)	72	86.57%	76.55%	14.47	3.74
MC-Net [3]	8 (10%)	72	87.71%	78.31%	9.36	2.18
ISO-MT (ours)	8 (10%)	72	87.93%	78.85%	7.93	2.07
V-Net	16 (20%)	64	86.03%	76.06%	14.26	3.51
UA-MT-UN [2]	16 (20%)	64	88.83%	80.13%	10.04	3.12
UA-MT [2]	16 (20%)	64	88.88%	80.21%	7.32	2.26
SASSNet [21]	16 (20%)	64	89.54%	81.24%	8.24	2.20
DUWM [22]	16 (20%)	64	89.65%	81.35%	7.04	2.03
LG-ER-MT [32]	16 (20%)	64	89.62%	81.31%	7.16	2.06
DTC [20]	16 (20%)	64	89.42%	80.98%	7.32	2.10
MC-Net [3]	16 (20%)	64	90.34%	82.48%	6.00	1.77
ISO-MT (ours)	16 (20%)	64	90.85%	83.38%	5.23	1.55

**Table 2:** Ablation experiments, where v stands for voxel.

Method	# Scans Used		Metrics			
	Labeled	Unlabeled	Dice	Jaccard	95HD (v)	ASD (v)
Teacher1	8 (10%)	72	86.87%	77.95%	7.40	1.95
Teacher1 + PO	8 (10%)	72	87.75%	78.65%	7.67	2.20
Teacher1 + teacher2	8 (10%)	72	87.70%	78.63%	7.66	1.96
Teacher1 + P + Teacher2	8 (10%)	72	87.93%	78.85%	7.93	2.07
Teacher1	16 (20%)	64	90.19%	82.27%	6.56	2.01
Teacher1 + PO	16 (20%)	64	90.65%	83.03%	5.16	1.67
Teacher1 + teacher2	16 (20%)	64	90.44%	82.84%	5.19	1.63
Teacher1 + PO + teacher2	16 (20%)	64	90.85%	83.38%	5.23	1.55

## Data Availability

Not applicable.
